# A Review of Biomarkers for Ischemic Stroke Evaluation in Patients With Non-valvular Atrial Fibrillation

**DOI:** 10.3389/fcvm.2021.682538

**Published:** 2021-07-01

**Authors:** Luxiang Shang, Ling Zhang, Yankai Guo, Huaxin Sun, Xiaoxue Zhang, Yakun Bo, Xianhui Zhou, Baopeng Tang

**Affiliations:** ^1^Department of Cardiology, The First Affiliated Hospital of Shandong First Medical University & Shandong Provincial Qianfoshan Hospital, Shandong Medicine and Health Key Laboratory of Cardiac Electrophysiology and Arrhythmia, Jinan, China; ^2^Xinjiang Key Laboratory of Cardiac Electrophysiology and Remodeling, The First Affiliated Hospital of Xinjiang Medical University, Urumqi, China; ^3^Department of Pacing and Electrophysiology, The First Affiliated Hospital of Xinjiang Medical University, Urumqi, China

**Keywords:** atrial fibrillation, non-valvular atrial fibrillation, ischemic stroke, biomarker, CHA2DS2-VASc score

## Abstract

Atrial fibrillation (AF) is the most prevalent cardiac arrhythmia worldwide and results in a significantly increased ischemic stroke (IS) risk. IS risk stratification tools are widely being applied to guide anticoagulation treatment decisions and duration in patients with non-valvular AF (NVAF). The CHA_2_DS_2_-VASc score is largely validated and currently recommended by renowned guidelines. However, this score is heavily dependent on age, sex, and comorbidities, and exhibits only moderate predictive power. Finding effective and validated clinical biomarkers to assist in personalized IS risk evaluation has become one of the promising directions in the prevention and treatment of NVAF. A number of studies in recent years have explored differentially expressed biomarkers in NVAF patients with and without IS, and the potential role of various biomarkers for prediction or early diagnosis of IS in patients with NVAF. In this review, we describe the clinical application and utility of AF characteristics, cardiac imaging and electrocardiogram markers, arterial stiffness and atherosclerosis-related markers, circulating biomarkers, and novel genetic markers in IS diagnosis and management of patients with NVAF. We conclude that at present, there is no consensus understanding of a desirable biomarker for IS risk stratification in NVAF, and enrolling these biomarkers into extant models also remains challenging. Further prospective cohorts and trials are needed to integrate various clinical risk factors and biomarkers to optimize IS prediction in patients with NVAF. However, we believe that the growing insight into molecular mechanisms and in-depth understanding of existing and emerging biomarkers may further improve the IS risk identification and guide anticoagulation therapy in patients with NVAF.

## Introduction

Atrial fibrillation (AF) is the most prevalent cardiac arrhythmia in clinical practice (1). Results from the famous Framingham Heart Study and Atherosclerosis Risk in Communities (ARIC) cohort showed that the lifetime risk to develop AF was up to one in three (2, 3). It is estimated that AF will affect >8 million people in America by 2050, and 18 million people in Europe by 2060 (1). Hence, AF poses a markedly increasing burden worldwide. Meanwhile, AF is a well-recognized risk factor for ischemic stroke (IS), heart failure (HF), cognitive decline, and is associated with substantial morbidity, disability, and mortality (4). The risk of IS among patients with AF is ~5% per year and is up to 5-fold higher than the general population (5). AF is reported to contribute to almost 15–20% of all stroke cases, and AF-related stroke has higher mortality and permanent disability than strokes from other etiologies (6). Therefore, IS prevention is the central pillar of AF management.

Oral anticoagulants effectively prevent IS and improve outcomes among patients with AF (7). However, prior to anticoagulation, stroke risk assessment is the first and the most vital step to maximize the benefits of anticoagulant drugs. Clinicians should identify patients at high-risk for IS, who will benefit in the first line from anticoagulation, or rather determine patients at low-risk of IS, in whom anticoagulation may not be warranted. In current clinical practice, the CHA_2_DS_2_-VASc score is recommended by the most influential guidelines as the primary means of stratifying patients with non-valvular AF (NVAF) (8–10). The major advantage of the CHA_2_DS_2_-VASc score is its perspicuity and simplicity of use, as it is a clinical risk-factor-based prediction score. However, it also has several drawbacks, such as widely ranged stroke rates of non-anticoagulated AF patients in different populations, and a limited predictive ability of stroke events (11, 12).

In recent years, biological markers (biomarkers) have been constituted a very powerful tool in the early diagnosis, risk stratification, prognosis prediction, and guiding therapy in many cardiovascular diseases (13, 14). According to the definition of the Biomarkers Definitions Working Group, “any characteristic that can be objectively measured and evaluated as an indicator of normal biological processes, pathogenic processes, or pharmacologic responses to a therapeutic intervention” is belonged to a biomarker (15). With the advancements in medicine, the contents of biomarkers have also continuously extended. A number of studies have explored differentially expressed biomarkers in NVAF patients with and without IS, and the potential role of various biomarkers for the prediction or early diagnosis of IS in patients with NVAF. Some previous publications have also summarized these biomarkers (16–21). However, in recent years, research into the possible biomarkers capable of predicting the IS events in patients with NVAF is constantly growing. Hence, our present updated review will focus on the current status of clinical biomarkers beyond the CHA_2_DS_2_-VASc score for the assessment of IS in patients with NVAF, which might provide a basis for the future perspectives of clinical application.

## CHA_2_DS_2_-VASc Score and Its Limitations

In 2001, Gage et al. created the CHADS_2_ index that included five variables: congestive HF, hypertension, age, diabetes, and stroke, for a maximum of 6 points, and has been well-validated in the National Registry of AF, which showed high prediction performance (c-statistic of 0.82) (22). However, later studies indicated that a CHADS_2_ score of 0–1 has poor identification of NVAF patients at truly low risk of IS (23, 24). Moreover, this score ignored several potential clinical risk factors for IS. Thus, in 2010, the CHA_2_DS_2_-VASc score was developed by re-stratifying the risk of IS based on the CHADS_2_ score, which incorporated three additional components: vascular disease, age 65–74, and female sex (25). A national prospective study has confirmed that the predictive ability for low risk of IS with the CHA_2_DS_2_-VASc score is significantly superior to the CHADS_2_ score, which provides more reliable guidance to determine whether or not anticoagulation treatment is required in patients with NVAF (23). The above two scores exhibited similar predictabilities in meta-analytic data, but CHA_2_DS_2_-VASc score had the important advantage of identifying extremely low-risk patients (26). Altogether, the CHA_2_DS_2_-VASc score is currently considered as a core risk stratification model for IS assessment in patients with NVAF.

Despite the simplicity and practicality, certain limitations exist in the CHA_2_DS_2_-VASc score. First, the contribution of the individual component to the risk of IS in patients with NVAF is unequal, but most components carry equal weight, and only two risk factors, age and prior stroke/transient ischemic attack (TIA), are assigned with different points (27, 28). Second, cardiovascular complications screening varies in practice by country and region. For example, ankle-brachial index (ABI), an indicator of peripheral arterial disease (PAD), is not routinely assessed in developing countries, which might potentially lead to an underestimation of the overall IS risk. Third, racial/ethnic differences may exist in IS risk prediction in NVAF, and the CHA_2_DS_2_-VASc score may not be validated in an ethnically diverse population (29). For example, the determination of the age threshold of IS risk assessment may vary with different populations (30). Forth, several other identified risk factors or biomarkers not included in the score, which lead to a suboptimal predictive performance in selected populations (e.g., patients with renal insufficiency) (31). Moreover, evidence from a recent systematic review shows that this score has not ideal predictive power (c-statistic of 0.6–0.7) (12). It is, thus, essential to improve the prediction accuracy of this model.

## Other Risk Stratification Models

Several recent studies have attempted to refine the CHA_2_DS_2_-VASc score system. Maheshwari et al. found that abnormal P-wave axis (aPWI) can predict the occurrence of IS independent of CHA_2_DS_2_-VASc variables, and proposed P_2_-CHA_2_DS_2_-VASc score to assess the risk of AF-related stroke, in which aPWI was scored with 2 points (32). A study from China indicated that urine albumin was an independent predictor of thromboembolism (TE) events for NVAF patients, and the new CHA_2_DS_2_-VASc-UA_2_ score system showed better performance in predicting TE events compared with the CHA_2_DS_2_-VASc score (33). Similarly, an analysis of the national health insurance database of more than 460,000 AF patients shows that the addition of African-American race (1 point) to CHA_2_DS_2_-VASc score (CHA_2_DS_2_-VASc-R score) significantly improved stroke prediction (34). In a recent study analyzing Korean NVAF populations, chronic kidney disease (CKD) but not female sex is an independent predictor of TE events (35). The authors proposed a CHA_2_DS_2_-VAK score in which “S”ex “c”ategory was replaced by “K”idney disease, the new score system enhanced discrimination of low to intermediate TE risk in NVAF patients (35). In another Asian study, a modified mCHA_2_DS_2_-VASc score, which assigned one point for patients aged 50–74 years, demonstrated a better predictive performance than the CHA_2_DS_2_-VASc in Taiwan AF population (36). Patients with a mCHA_2_DS_2_-VASc score of 1 (males) or 2 (females) obtained positive net clinical payoffs from anticoagulation therapy (36).

Furthermore, investigators are also proposing new prognostic models, such as ATRIA score, GARFIELD-AF model, and ABC stroke score (Table 1). Similar to the CHA_2_DS_2_-VASc score, these models all incorporate age and common clinical risk factors of IS. The ATRIA score takes into account the interaction between age and previous stroke (37). The GARFIELD-AF model is composed of more than 30 clinical risk factors (38). The ABC stroke risk score is a biomarkers-based nomogram, which include age, N-terminal prohormone of brain natriuretic peptide (NT-proBNP), cardiac troponin I (cTnI), and prior stroke/TIA (39). Therefore, the calculation of these scores is complicated, making it impractical for clinical use. Moreover, a recently published systematic review, which summarized current risk stratification tools for IS prediction in patients with NVAF, did not show a better prediction role for the above scores in improving the ability of IS events compared with CHADS_2_ and CHA_2_DS_2_-VASc score (12). Taken together, despite the limitations of the CHA_2_DS_2_-VASc score, no strong evidence has been able to show that these novel or modified risk scores can replace it.

**Table 1:** Influential IS risk stratification models/scores for NVAF.

**Model/Score**	**Components**	**Points**	**Range of stroke risk stratification**	**Validation studies**
CHADS_2_ score	Heart failure, hypertension, age, diabetes, stroke	0 to 6	Low (0 point), moderate (1 point), high (≥2 points)	Yes
CHA_2_DS_2_-VASc score	Heart failure, hypertension, age≥75, diabetes, stroke, vascular disease, age 65–74, female sex	0 to 9	Low (0 point), moderate (1 point), high (≥2 points)	Yes
ATRIA score	Age, prior stroke, female sex, diabetes, heart failure, hypertension, proteinuria, eGFR <45 or ESRD	0 to 15	Low (0–5 points), moderate (6 points), high (7–15 points)	Yes
GARFIELD-AF model	Age, pulse, systolic blood pressure, vascular disease, history of bleeding, heart failure, renal disease, use of OAC^*^	Machine learning model	—	Yes
ABC stroke score	Age, prior stroke/transient ischemic attack, NT-proBNP, cTnI	Nomogram	—	Yes

* *Components of the simplified GARFIELD-AF risk model*.

*IS, ischemic stroke; NVAF, non-valvular atrial fibrillation; eGFR, estimated glomerular filtration rate; ESRD, end-stage renal disease; OAC, oral anticoagulation; NT-proBNP, N-terminal prohormone of brain natriuretic peptide; cTnI, cardiac troponin I*.

## Clinical Biomarkers Beyond the CHA_2_DS_2_-VASc Score for Is Evaluation in NVAF

**Figure 1:**
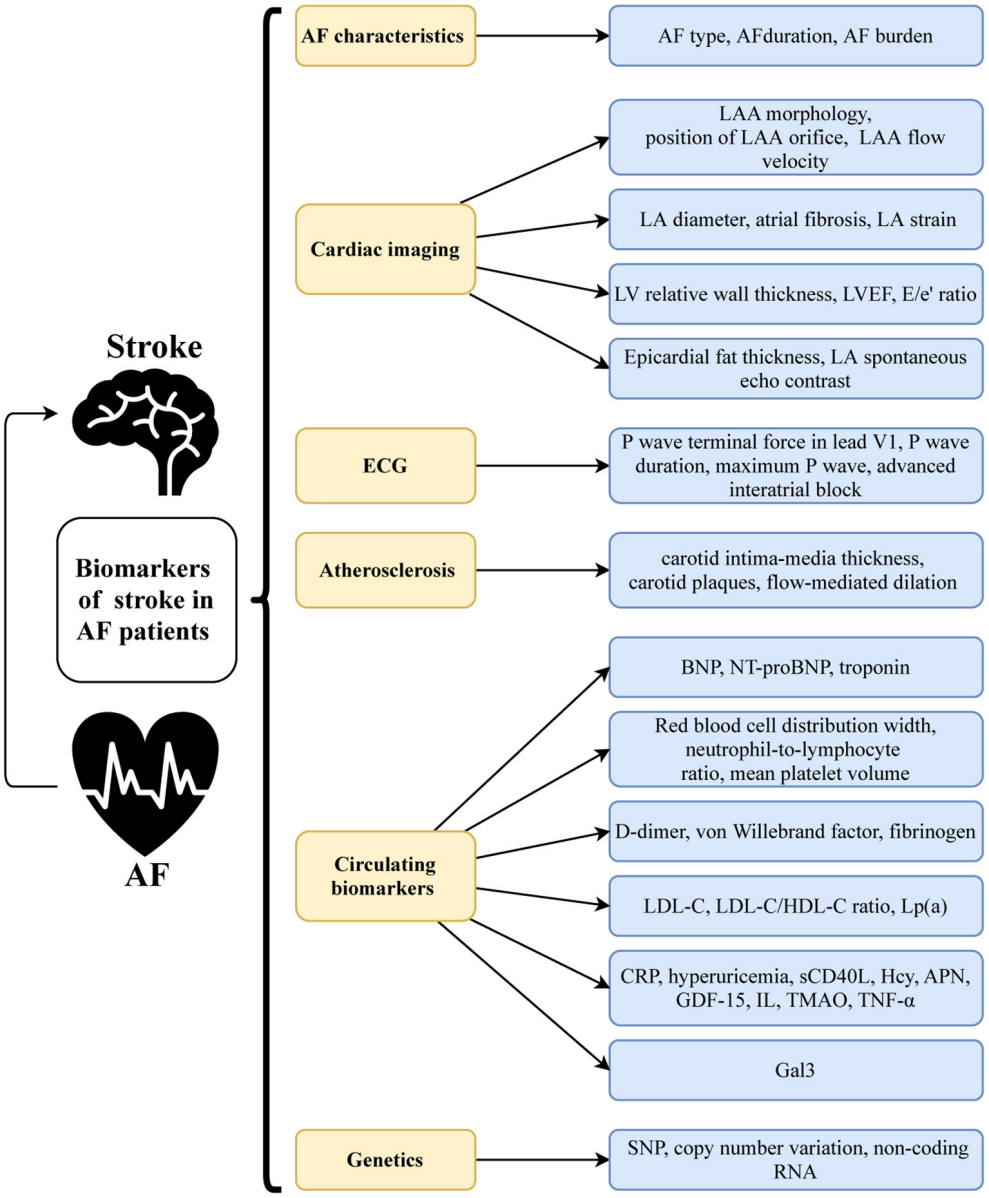
Classification of biomarkers in AF-related stroke.

While IS risk prediction scores have been heavily weighted by well-established clinical factors, findings from randomized controlled trials (RCTs) and community-based cohorts showed that various biomarkers can improve predictive accuracy and risk assessment. Numerous studies have examined the utility of AF characteristics, cardiac imaging and electrocardiogram (ECG) markers, atherosclerosis-related markers, circulating biomarkers, and novel genetic markers (Figure 1) in IS prediction in patients with NVAF. These biomarkers, whether new or old, may enhance our understanding of the pathophysiology of AF-related IS and help us to find new therapeutic targets.

## AF Characteristics

The type, duration, and burden of AF are the most frequent clinical characteristics assessed by clinicians. AF has been conventionally categorized into “valvular” or “non-valvular” on the basis of the presence or absence of valvular heart disease. Besides, the types of AF can be classified as first diagnosed, paroxysmal, persistent, long-standing persistent, and permanent AF in light of the presentation, duration, and spontaneous termination of AF episodes. The secondary analysis of several RCTs which examined the clinical efficacy of novel oral anticoagulants or aspirin in AF patients demonstrated that persistent and permanent AF increased the risk of IS compared to paroxysmal AF in patients taking anticoagulation therapy, as well as patients taking antiplatelet therapy (40–43). Similarly, in one Japanese cohort study, a lower incidence of stroke/systemic embolism was observed in paroxysmal AF compared with sustained AF regardless of oral anticoagulant uses (44). A meta-analysis of 12 studies containing 99,996 patients showed that non-paroxysmal AF is associated with an increase in TE events [hazard ratio (HR) = 1.384, 95% confidence intervals (CI):1.191–1.608, *P* < 0.001] compared with paroxysmal AF, and in subgroup analyses, this difference was present for both patients on oral anticoagulants and not on oral anticoagulants (45).

Although ECG and 24-h Holter are commonly applied in patients with AF, it still requires a longer period of continuous monitoring to obtain AF burden and duration. Current studies that assess the burden of AF and stroke risk are mostly based on patients with cardiovascular implantable electronic device (CIED) implantation. Atrial high rate episodes (AHREs) were exactly described as the unknown AF with a fast atrial episode (>180 bpm) recorded on CIED for at least 5 min (46). Epidemiological data reported the incidence of AHRE reached ~25–35% during 2-year follow-up in patients without a natural history of AF (47). Current evidence supported the elevated AHRE burdens increased the risk of adverse cardiovascular prognoses such as myocardial infarction, HF, and ventricular arrhythmia (48). Additionally, the association was being drawn between AHRE and increased stroke risk by a growing number of clinical trials. Early in 2003, the MOST (Mode Selection Trial) investigators prospectively evaluated the association between AHREs and clinical outcomes in sinus node dysfunction patients with pacemaker therapy. After the adjustments of prognostic and baseline variables, AHRE was reported as the independent risk factor of death or non-fatal stroke (HR = 2.79, 95% CI:1.51–5.15, *P* = 0.0092) by Cox proportional hazards analysis (49). The ASSERT trial on the larger sample size detected the AHREs in the population without diagnostic AF (*n* = 2,580) for 3 months after ICD implantation. Such subclinical AF, a confirmed predictor of stroke, contributed to the increased risk of IS or systemic embolism (HR = 2.49, 95%CI:1.28–4.85, *P* = 0.008) (50). Meanwhile, the correlation between asymptomatic AF and high TE risk has been illustrated by the EORP-AF Pilot General Registry (51). Compared with healthy controls, asymptomatic AF patients potentially progressed to permanent AF, and may lead to higher systemic ischemic events (52, 53). A proof-of-concept study found that machine-learned signatures of AF burden could provide prognostic information on the near-term risk of stroke in patients with CIED (54). However, a recent cohort study that included 384 CIED implanted patients without anticoagulation showed that the burden and duration of AF were not associated with IS/TIA, and only the CHA_2_DS_2_-VASc score can predict IS/TIA (55). The inconsistency in the aforementioned results may be caused by the difficult screening of subclinical ischemic brain lesions (IBLs), which results in an underestimated embolic rate. A Spanish research group prospectively assessed the relationship of AHRE and IBLs through the computed tomography scan in patients with CIED implantations, and results showed that AHRE was an independent predictor for silent IBL both in the overall population and in patients without a history of AF or stroke (56). Similar results were observed in patients with cardiac resynchronization therapy (57). However, there is no sufficient evidence to reveal the distinct temporal relevance between AHRE and subsequent events. As shown in TRENDS study, an AHRE episode is able to be recorded before, during, or after the stroke event (58). Based on current cognition, whether the AHRE performs a cause or merely a biomarker of TE should be interpreted more prudently. In addition, with the development of science and technology, the increasing using of wearable devices and apps in daily life and clinical practice may be useful and convenient to quantify AF burden (59). We consider that more research is needed in the future to explore the role of wearables-detected AF burden in evaluating IS events.

## Cardiac Imaging and ECG Biomarkers

According to Virchow's triad, there are three pivotal factors to venous thrombosis: vascular damage, blood stasis, and hypercoagulability. With the progression of AF, progressive atrial dilatation, endocardial denudation, and oedematous or fibroelastic infiltration of the extracellular matrix will lead to abnormal blood flow patterns through the atrium and the formation of intra-atrial thrombus (60). Therefore, using parameters that reflect cardiac structural and functional remodeling to predict the risk stratification of TE events in NVAF is very meaningful. In fact, a large number of studies have investigated the role of cardiac, especially atrial structure, function, electrocardiography, and cardiac circulating biomarkers in AF-related IS.

### Left Atrial Appendage (LAA) Structure and Function

LAA is an embryological remnant of the primordial left atrium (LA). As early as two decades ago, LAA was reported to be closely related to atrial thrombus formation in NVAF patients because of its hooked morphology and “low flow state” (61). In 2012, Di Biase et al. (62) firstly divided LAA morphology into four types: Chicken wing, Cactus, Windsock, and Cauliflower, and reported that NVAF patients with non-Chicken Wing (CW) LAA morphologies were more likely to occur TE events than CW patients after controlling for comorbidities and CHADS_2_ score. A later meta-analysis included in 12 studies showed that the risk of cerebrovascular accident in AF patients with CW morphology was reduced by 41% relative to non-CW patients (63). However, this subjective classification of LAA morphology is not well-quantifiable and can be widely influenced by clinicians and reviewers. In one study conducted in Fuwai Hospital, the consensus of LAA morphology was only reached in 28.9% among three experienced reviewers (64). A retrospective study revealed that the classification of with or without clearly lobulated structure of LAA, a relatively concise classification strategy, was independently associated with LAA thrombosis in NVAF patients (OR = 4.216, 95% CI: 1.825–9.740, *P* = 0.001) (65). Several recent studies investigated the role of quantitative assessments of LAA, such as the angle bend from the proximal/middle portion of the LAA (66) and statistical shape analysis of LAA (67) in predicting stroke. Nevertheless, large-scale validation is needed to further verify these preliminary findings. Additionally, Zhao et al. (68) showed that higher position of LAA orifice had a strong relationship with thrombus formation after adjusting for confounding factors in AF patients, which was consisting with Nedios et al. (69), who reported that higher position of the superior LAA-takeoff in NVAF patients was paralleled with increasing TE events after catheter ablation. Furthermore, the Stroke Prevention in Atrial Fibrillation (SPAF-III) Study trial revealed that low LAA flow velocity (<0.2 m/s), reflecting the systolic function of LAA, was associated with TE events in AF patients (70). Similar results were shown in a Korean study, in which increased orifice size and decreased flow velocity of LAA were related to IS risk in patients with NVAF (71). In a word, the structural and functional characteristics of LAA contributed to the assessment of IS risk in NVAF patients.

### LA Structure and Function

The structure and function of LA could also assist in IS prediction in patients with NVAF (72, 73). In Fushimi AF Registry, a large community-based cohort study of Japanese NVAF patients, larger LA diameter (LAD) was a strong predictor of stroke/TE whether oral anticoagulant was used or not (HR = 1.74, 95% CI: 1.25–2.42, *P* < 0.01) (74). This was paralleled with the result from the Framingham Heart Study (75), in which LA enlargement remained a significant predictor of stroke in male AF patients. Paciaroni et al. (76) reported that severe LA dilation (defined by LAD ≥ 5.0 cm/m^2^ or LAVi ≥ 40 ml/m^2^) was associated with the incidence of TE events (OR = 2.05, 95% CI: 1.08–2.87, *P* = 0.027). Additionally, the functional status of LA also needs to pay more attention. It has been established that atrial fibrosis, which could be assessed with late gadolinium enhancement (LGE) on cardiac magnetic resonance imaging (MRI), was independently associated with the higher risk of TE events (77, 78). In a mean of 7.9 years follow-up study of 1,361 first diagnosis of AF patients, P-wave to A′ duration on tissue Doppler imaging, reflecting total atrial conduction time, was independently associated with IS risk in a fully-adjusted model including CHA_2_DS_2_-VASc score, age, and anticoagulant use (79). Furthermore, previous studies have documented that LA strain was also associated with LA fibrosis, and the independent relationship between the reduced LA strain, strain rate, and IS were subsequently certificated (80–82).

### Left Ventricular (LV) Structure and Function

Parameters of LV structure have shown closely related to stroke events in AF patients, the underlying pathophysiologic mechanism lies in that elevated LV filling pressure would lead to LV hypertrophy and subsequent LA dilation (73). In the ARAPACIS Study, the prevalence of LV hypertrophy in patients with NVAF is higher, which is consistent with the higher risk of TE risk in these patients (83). Meantime, in a large community-based prospective study, Tezuka et al. (84) manifested that after adjustment for various potential confounders, high LV relative wall thickness (RWT) was independently associated IS in NVAF patients (HR = 1.81, 95% CI: 1.34–2.47, *P* < 0.01), indicating the vital role of LV morphology in contributing to TE. Additionally, LV systolic function categorized by LV ejection fraction (LVEF) was thought of as a key TE event predictor in NVAF. In fact, as the most important diagnostic indicator of HF, LVEF has already been included in the CHA_2_DS_2_-VASc score. In 1992, Asinger et al. (85) found that LV dysfunction was associated with TE events in 568 AF patients (RR = 2.0, 95% CI: 1.0–4.0, *P* < 0.05). Result from 3 RCTs including 1,066 NVAF patients also showed that moderate to severe LV systolic dysfunction was a strong independent predictor of stroke (RR = 2.5, 95% CI: 1.5–4.4, *P* < 0.01) (86). As a more reliable and stable index of LV dysfunction, we speculate that LV strain could provide a significant advantage in predicting IS compared with LVEF. However, as far as we know, there was no current study certificated that the LV strain could be used as a predictor of stroke in NVAF. Besides, previous studies have documented the evident relationship between chronic LV diastolic dysfunction and LA enlargement, promoted the AF occurrence and thrombi formation (73). Among LV diastolic parameters, E/e′ ratio had an independent association with stroke in NVAF patients (OR = 1.21, 95% CI:1.08–1.37, *P* = 0.002) (87). Therefore, structural and functional parameters of LV also play an important role in predicting IS in patients with NVAF.

### Other Echocardiographic Indicators

In addition to the above markers, other echocardiographic indicators also deserve considerable attention. In a matched cross-sectional study, the role of epicardial fat thickness in AF patients with and without acute IS was analyzed, and higher epicardial fat thickness (OR = 7.356, 95% CI: 3.880–13.947, *P* < 0.0001) independently predicted acute IS (88). Early studies demonstrated that LA spontaneous echo contrast (LASEC), a frequent finding on transesophageal echocardiography, was thought to a marker of the hypercoagulable state (89). A prospective cohort study showed that NVAF patients with IS had higher grades and video intensity value of LASEC compared with patients without IS, and the video intensity value of LASEC had a better predictive performance of IS in NVAF patients than LA thrombus, CHADS_2_ score, and CHA_2_DS_2_-VASc score (90).

### ECG Markers

The P wave results from electric activity in the atrium and is an indicator of atrial depolarization (91). Thus, P wave indices could be used to evaluate the LA abnormalities, which might further be associated with increased risk of IS in AF patients. A recent systematic review reported that several common P wave indices, including P wave terminal force in lead V1, P wave duration, and maximum P wave area were predictors of IS (92). As earlier mentioned, an abnormal P-wave axis was associated with increased IS risk independent of CHA_2_DS_2_-VASc score in AF patients (32). In addition, some small sample studies have shown that advanced interatrial block, diagnosed upon the duration of the P wave and morphology in limb lead ECG, could serve as a marker of atrial electromechanical dysfunction and a surrogate for LA strain reduction, and might act as a predictor of IS in AF patients (93, 94).

In summary, parameters of cardiac MRI, echocardiography, and ECG could reflect the atrial and ventricular structure and function and could be used as risk predictors of IS in NVAF. Moreover, in the last years, innovations in multi-modality imaging can offer a comprehensive evaluation of cardiac remodeling, which we believe could be further used for accurate IS prediction in AF patients (95).

## Arterial Stiffness and Atherosclerosis-Related Markers

Atherosclerosis a well-recognized risk factor for IS in the general population. Vascular diseases, including a history of myocardial infarction, aortic plaque, and PAD, is a component of the CHA_2_DS_2_-VASc score. However, as previously mentioned, PAD and aortic plaques examinations are not routinely performed for AF patients in many instances. Therefore, many studies have explored the predictive role of other arteriosclerosis-related indicators such as carotid intima-media thickness (cIMT), carotid plaques, and flow-mediated dilation (FMD) with IS in NVAF.

Our previous hospital-based study showed that carotid plaque was detected in more than half of patients with NVAF (96). In fact, a growing number of studies across the globe have shown that carotid plaque is more common in patients with AF than in those without AF (97). Result from ARAPACIS study showed that the combination of vascular diseases and carotid plaque was independently associated with stroke in patients with NVAF (HR = 1.78, 95% CI: 1.05–3.01, *P* = 0.0318) (98). Similarly, two studies from Korea (99) and the USA (100) both demonstrated that the addition of cIMT and carotid plaque in the CHA_2_DS_2_-VASc score can better predict the occurrence of IS in patients with AF. In addition, a case-control study suggested that the stability of carotid plaques was also associated with IS in patients with NVAF (101). A recently published systematic review identified available data and confirmed an association of carotid atherosclerosis with the risk of IS and TIA in patients with AF (102). In a 2-year follow-up study, low FMD was associated with an increased composite endpoint for cardiovascular events including IS in NVAF patients (103). It is noteworthy that evidence from a meta-analysis revealed that the use of statins, the most common clinically anti-atherosclerotic agent, reduces mortality in AF patients, which might, on the other hand, illustrate that atherosclerosis has a detrimental role contributing to IS in patients with AF (104). In general, given its simplicity and stability, we believe that carotid plaque is a promising marker for improving classification in CHA_2_DS_2_-VASc score in NVAF patients.

## Circulating Biomarkers

### Cardiac Biomarkers

There is a broad consensus that elevated B-type natriuretic peptide (BNP) and NT-proBNP are the hallmarks of HF (105). BNP is mainly produced by cardiac myocytes as a response to increased end-diastolic pressure and/or volume expansion, and then enzymatically cleaved to the NT-proBNP (106). Recent studies suggested that BNP and NT-proBNP might also have the effect of predicting stroke in AF patients. One possible reason is that the increased pressure of atrial myocytes can lead to the increased secretion of BNP, and thus reflecting atrial dysfunction (16). An early small-sample research indicated that elevated BNP level was significantly associated with TE events in AF patients treated with warfarin (107), since increased levels of BNP was observed at the acute stage of IS (108) and in patients with a history of TE or echocardiographic evidence of thrombus (109) of NVAF patients. *Post-hoc* analyses of ARISTOTLE trial (110), RE-LY trial (111), and ENGAGE AF-TIMI 48 trial (112) similarly showed that NT-proBNP was independently associated with the increased risk of IS, and adding NT-proBNP to the CHA_2_DS_2_-VASc score could improve C-statistics. Similarly, a single-center study showed that incorporating NT-proBNP into the CHA_2_DS_2_-VASc score increased the ability of IS/systemic embolism risk prediction in anticoagulated patients with AF by 17% (113). Result of the Hokuriku-Plus AF Registry illustrated that high levels of BNP were also increased the risk of TE events in NVAF patients (114), which was corresponded with the findings of Paulin et al. (115). Evidence from a multicenter, prospective observational study (Fushimi AF Registry) showed that BNP was associated with IS and TE events in patients with AF without HF, and the addition of BNP into CHA_2_DS_2_-VASc score as a new risk prediction model can better predict IS risk (116). In addition, high levels of BNP can be used as a predictive marker for recurrent IS in IS survivors with AF (117). Moreover, BNP can also be used as an etiological diagnosis indicator of acute IS in patients with AF. Sakamoto et al. (118) reported that low levels of BNP (<130 pg/mL) were associated with non-cardiogenic IS, while high levels of BNP were associated with cardiogenic IS, which may be due to the fact that BNP could promote intracardiac thrombosis.

Troponin is a marker of myocardial injury and is widely used in the diagnosis and prognosis of acute coronary syndrome (105). As the most widely applied biomarker in cardiovascular disease, emerging evidence suggested that troponin could also predict stroke in patients with AF. In a retrospective study of 199 NVAF patients, elevated hs-cTnI was independently associated with abnormal anatomy of the LA, defined as LAA flow velocity <20 cm/s or dense spontaneous echo contrast, and the incidence of IS increases with higher cTnI levels (119). Similar to NT-proBNP, the *post-hoc* analyses of ARISTOTLE trial (120), RE-LY trial (111), and ENGAGE AF-TIMI 48 trial (112) also indicated a positive association between cTnI/cTnT level and risk of TE/IS, and integrating troponin to the CHA_2_DS_2_-VASc score could improve C-statistics. In a real-world cohort study, after adjusting for CHA_2_DS_2_-VASc score, a high level of cTnT (≥8.04 ng/L) was shown to be associated with IS/TIA in patients with AF (HR = 2.44, 95%CI:1.13–5.26, *P* = 0.023) (121). In a study validating the performance of ABC risk score, cTnT has also been shown to be correlated with IS/systemic embolism in patients with AF (122). A recent meta-analysis which focused on the correlation between hs-cTnT and risk of stroke showed that the HR value of IS in AF patients with a high level of hs-cTn was 1.95 (95% CI: 1.29–2.62), suggesting that hs-cTn could be used as a marker of IS risk stratification in AF patients (123).

To sum up, the BNP, NT-proBNP, and cTn are shown to be effective to improve risk stratification in addition to the current CHA_2_DS_2_-VASc score. They are widely used and readily accessible in clinics, and are easy to popularize in daily clinical practice. Further, the dynamic evolution of each cardiac marker must not be overlooked. In a very recent result from ENGAGE AF-TIMI 48 trial, there were quite a large number of AF patients who experienced dynamic changes of NT-proBNP and hs-cTnT in the follow-up, and upward changes in these markers were associated with increased risk of IS/systemic TE (124). This might increase the burden of these markers on clinical application.

### Markers of Routine Blood Test

The complete blood cell count is one of the most frequently ordered laboratory tests in clinical practice. Previous findings suggested that several parameters in routine blood tests, such as red blood cell distribution width (RDW), neutrophil-to-lymphocyte ratio (NLR), and mean platelet volume (MPV) might relate to the evaluation of IS in patients with NVAF.

RDW is a quantitative measurement of differences in the size and volume of circulating red blood cells, and increased RDW reflects the existence of erythrocytopenia, caused by impaired erythropoiesis or erythrocyte degradation, which reflect underlying chronic inflammation and high levels of oxidative stress state (125). In a cross-sectional study, RDW was independent correlated with the increase of CHADS_2_ and CHA_2_DS_2_-VASc score in patients with NVAF, suggesting that RDW could predict the risk of TE (126). Likewise, a high RDW (>13.16%) was shown to be associated with LA thrombosis in patients with NVAF in another study (127). In an up-to 5.2-year follow-up “real-world” retrospective cohort study, increased RDW value was independently associated with TE events in patients with NVAF (128). The result of a national study showed that the cumulative stroke incidence in AF patients not taking anticoagulants at baseline increased across RDW quartiles, and after adjusting for known conventional clinical risk factors, RDW was independently associated with stroke (129).

NLR is a marker of systemic inflammation. Specifically, the high neutrophil count reflects subclinical inflammation, while the decrease of lymphocyte count reflects an impairment of the adaptive immune system and poor general health status (130). A preliminary study showed that NLR levels were significantly correlated with CHA_2_ DS_2_-VASc score in NVAF patients (131). A small sample study showed that the level of NLR in patients with NVAF complicated with IS was higher than that of non-stroke patients (132). A subsequent large sample cohort study further revealed that the incidence rate of stroke increased across NLR quartiles in patients with AF, and NLR refined the risk of stroke across all CHA_2_DS_2_-VASc score strata (133).

MPV is considered to indicate the intensity of the inflammatory process and risk of thrombotic complications (134). Emerging evidence also supports the use of MPV as a biomarker for predicting IS risk in patients with AF. An earlier study showed that MPV was a predictive marker for stroke in patients with AF; its predictive power for stroke was independent of age, gender, and other CHADS_2_ score components (135). In a study composed of 352 NVAF patients, high MPV was found to be an independent predictor of the composite of IS event and incidental LA thrombus (136). Gul et al. (137) found that MPV levels were significantly higher in acute IS patients with NVAF than those without NVAF. A study of NVAF patients who did not receive anticoagulant therapy showed that MPV was an independent predictor of IS in this population, and the combination of MPV and CHA_2_DS_2_-VASc score had improved predictive value and sensitivity, suggesting MPV could be used as a powerful tool for risk stratification of IS in patients with NVAF (138).

### Coagulation Markers

As a component of Virchow's triad, hypercoagulability is considered an integral mechanism in the pathogenesis of thrombosis (139). Therefore, indicators of coagulation tests might have potential value in predicting AF-related IS. Studies have shown that D-dimer, von Willebrand factor (vWF), and fibrinogen may become new therapeutic targets or auxiliary diagnostic means to assist the risk stratification in AF-related IS.

D-dimer is a specific degradation product of cross-linked fibrin, and a biomarker indicating the activation of coagulation and fibrinolysis (140). In a cross-sectional study, D-dimer levels were positively associated with LA enlargement in anticoagulation-naïve patients with an acute IS and NVAF, suggesting that D-dimer could be helpful as a potential surrogate and predictive marker for adverse cardiovascular events in NVAF patients (141). Sub-analysis of several large RCTs testing the efficacy of direct oral anticoagulants vs. warfarin showed that greater levels of D-dimer were associated with higher frequencies of IS or systemic TE events (112, 142, 143). In a study of 509 NVAF patients, D-dimer level in combination with clinical risk factors could effectively predict subsequent TE events even when treated with warfarin (144). In a prospective observational study, AF patients with high levels of D-dimer have increased an risk of composite cardiovascular endpoint (myocardial infarction, stroke or TIA, and arterial embolic events) (145). In a retrospective study, the correlational analysis revealed that D-dimer levels are directly related to stroke volume, severity, and prognosis in patients with NVAF (146). However, a study of 323 NVAF patients, who did not receive anticoagulant therapy, revealed that only the D-dimer level at stroke onset were independent risk factors for IS, while baseline D-dimer levels was not an independent risk factor for IS (147). Therefore, the dynamic detection of D-dimer levels might be necessary for patients with NVAF.

vWF is a plasma glycoprotein synthesized by endothelial cells during endothelial cell activation or injury, which promotes platelet adhesion and aggregation at the site of vascular injury, and is a definite marker of endothelial injury or dysfunction (148). Elevated vWF levels were found in patients with AF compared with healthy controls in an early study (149). A later cross-sectional study showed that raised plasma vWF was associated with four recognized risk factors for IS in AF patients (advancing age, prior IS, HF, and diabetes) (150). In a prospective study, plasma vWf levels were a significant predictor of stroke in NVAF patients taking aspirin, however, after adjustment for other clinical predictors, the relationship between vWf and stroke became non-significant (151). In a 3-year follow-up study, Pinto et al. (152) pointed that baseline vWF was a predictor of new-onset IS in patients with chronic NVAF. In another study over a median follow-up of 5.4 years, elevated plasma vWF was an independent risk factor for IS and all-cause death in patients with NVAF (153).

It has been already observed that the levels of fibrinogen were significantly higher in AF patients than in sinus rhythm patients (154). In patients with AF, those with a higher CHA_2_DS_2_-VASc score had increased fibrinogen level compared with those with a low risk of IS (155). Plasma fibrinogen level was associated with a history of stroke in NVAF patients in a case-control study (156). In addition, fibrinogen was also independently and positively associated with leukoaraiosis and periventricular hyperintensity in patients with stroke and AF (157).

### Lipid Markers

Dyslipidemia is closely associated with cardiovascular disease and is the important predictor and therapeutic target of cardiovascular risk. After optimizing the stratification of risk factors other than CHA_2_DS_2_-VASc scores, a recently published meta-analysis showed that the levels of low-density lipoprotein cholesterol (LDL-C) and total cholesterol in the IS group were higher than those in the non-stroke group in NVAF patients (28). A case-control study showed that LDL-C is an independent predictor of IS in patients with NVAF and could improve stroke risk stratification (158). However, another large sample retrospective cohort study did not reach a consistent conclusion. The study carried by Omelchenko et al. (159) showed that LDL-C levels were not associated with the risk of IS in NVAF patients treated with oral anticoagulants, and they interpreted this lack of association as the high selectivity of patients (patient taking oral anticoagulants) and the high proportion of TE etiology for IS in AF patients. On the other hand, compliance with statins was associated with a reduced risk of recurrent IS in patients with AF, suggesting that AF status should not be a condition for excluding statins as a condition for secondary stroke prevention in patients with IS (160). High-density lipoprotein cholesterol (HDL-C) is an anti-atherosclerotic lipoprotein and is reported negatively correlated with the risk of IS (161). Our previous case-control study has shown that the LDL-C/HDL-C ratio is a predictor of IS in patients with NVAF (162). Elevated LDL-C/HDL-C ratio may suggest that the imbalance between atherosclerotic and anti-atherosclerotic components, and the increase of pro-inflammatory components, which might both affect the occurrence and development of IS. In a small-sample study, NVAF patients with IS have higher levels of lipoprotein (a) [Lp(a)], and Lp(a) ≥30 mg/dL is associated with TE events in patients with NVAF (163). However, results from ARIC cohort showed that high Lp(a) levels were associated with increased IS risk, primarily among individuals without AF but not in those with AF (164). Overall, at present, the results of the relevant observational studies on blood lipids and IS in patients with NVAF are contradictory. High-quality evidence is lacking. Further studies are still needed to confirm the relationship between blood lipids and IS in patients with NVAF.

### Oxidative Stress and Inflammation Biomarker

Oxidative stress and inflammation are tightly linked to AF (165). Therefore, markers of inflammation might be identified as predictors of AF-related IS. Numbers studies have shown that various inflammation markers, such as C-reactive protein (CRP), hyperuricemia, soluble CD40 ligand (sCD40L), homocysteine (Hcy), adiponectin (APN), growth differentiation factor 15 (GDF-15), circulating interleukins (IL) might be useful biomarkers for predicting AF-related IS.

CRP is the most commonly used measure of the inflammatory response. A high CRP level was associated with LA enlargement and depression of contractile function of LA in paroxysmal AF patients (166). Secondary analysis of SPAF III clinical trial (167) and RE-LY trial (168) showed that CRP was positively correlated to stroke risk in AF patients taking aspirin or oral anticoagulant.

Hyperuricemia is a known independent competing risk factor for AF (169). Recent studies also demonstrated that hyperuricemia was associated with IS among AF patients. Several case-control studies showed that uric acid level was closely associated with LA stasis (composed of LA thrombus, LASEC) in patients with NVAF (170–173). In a study of NVAF patients at clinically low-intermediate risk (CHA_2_DS_2_-VASc score = 0 or 1), uric acid levels were higher in those with transesophageal echocardiography (TEE) thromboembolic risk than in those without TEE risk (174). Hyperuricemia was shown to dependently predict IS after adjusting for CHA_2_DS_2_-VASc score and other comorbidities in a cohort study, and could further stratify low-risk patients into 2 groups with different stroke rates (175).

sCD40L has been considered as a marker of thrombosis and inflammation in several diseases. In patients with AF, the presence of LA thrombus was associated with significantly increased levels of sCD40L (176). In a study of 44 consecutive outpatients with chronic NVAF, plasma sCD40L was the independent variable for LASEC or LA thrombus formation, and for cerebrovascular events (177). Another larger study came to a similar conclusion, which enhanced soluble CD40L level was a predictor of fatal and non-fatal IS in patients with NVAF (178).

In an early study, hyperhomocysteinemia is associated with the presence of LA thrombus in stroke patients with NVAF (179). A later study showed that increased fasting Hcy levels were independently associated with a history of IS in NVAF patients hospitalized for cardiac reasons (180). AF and elderly patients were shown to have elevated Hcy levels, which might result in the correlation between high levels of Hcy and stroke in the elderly AF patients (181).

APN possesses anti-inflammatory and antiatherogenic effects. In a cross-sectional study, APN levels were higher in anticoagulated AF patients with LASEC, a LA thrombus, or a LAA thrombus (182). Additionally, AF patients at high risk of stroke disclosed low levels of APN (183). However, in a study of 918 stable anticoagulated outpatients with NVAF, APN was neither predictive of stroke/TE in both male and female patients (184).

GDF-15 is a peptide hormone and a divergent member of the transforming growth factor-beta superfamily (185). In a cross-sectional study, elevated GDF-15 was associated with the presence of LA/LAA thrombus in NVAF patients without anticoagulation (186). Insights from ARISTOTLE trial (187) and ENGAGE AF-TIMI 48 (122) also showed that GDF-15 was a risk factor for stroke in AF patients with anticoagulation therapy.

At present, researchers are also focusing on the correlation between other markers of inflammation and AF-related IS. In a recent pilot study, trimethylamine N-oxide (TMAO) was an independent predictor in IS in AF patients, and the level of TMAO was correlated with the CHA_2_DS_2_-VASc score (188). Results of a two-sample Mendelian randomization study showed a positive association of IL-1ra with cardioembolic stroke and inverse associations of IL-6 with cardioembolic stroke (189). In a 3-year follow-up study, baseline plasma levels of TNF-α and IL-6 are predictors of new-onset IS at follow-up in patients with chronic NVAF (152). In addition, evidence from a meta-analysis showed that increased circulating plasminogen activator inhibitor-1 and thrombin-antithrombin levels were significantly associated with subsequent stroke in patients with AF (190).

In general, many studies have shown that indicators of inflammatory could predict stroke in NVAF patients. However, these biomarkers are diverse and lack specificity. Therefore, more research is needed to find reliable inflammatory markers.

### Fibrosis Markers

Cardiac (especially atrial) fibrosis is a critical feature of myocardial remodeling. The imaging manifestations of cardiac fibrosis, such as increased LAD, LA strain, and LGE, have been confirmed to be associated with an increased risk of IS in patients with AF as described previously. Several studies have also shown that circulating fibrosis biomarkers are associated with AF-related IS. High Gal-3 level was closely related to LAA flow velocity and occurrence of LAA thrombus in patients with NVAF (191). However, peripheral levels of circulating fibrosis biomarkers are susceptible to non-cardiac fibrosis, and might not be representative of the severity of cardiac fibrosis (192). Therefore, more research is required to explore the usefulness of circulating fibrosis in predicting AF-related IS in the future.

## Novel Markers of Genetics and Bioinformatics

At present, the research field of genetics and bioinformatics, and their applications to AF continue to evolve rapidly (193). Existing studies have identified a variety of genetic markers of AF-related IS through single nucleotide polymorphisms (SNP) analysis, genome-wide association study (GWAS), bioinformatic analysis, and omics. In an early study, a genetic risk score of twelve SNPs could identify individuals at increased risk for future AF and stroke (194). Based on the discovery of GWAS, copy number variation and SNPs could be genetic predictors of risk of TE and cardioembolic stroke for patients with AF (195–197). In a recent update study using data from the largest available GWAS in Europeans, a polygenic risk score incorporated of over half a million genetic variants could significantly improve net reclassification compared with CHA_2_DS_2_-VASc score in predicting IS in patients with AF (198). Two studies analyzed datasets of Gene Expression Omnibus via bioinformatic analysis, respectively, and identified several genes which were involved in AF-related stroke (199, 200). In addition, studies have shown that abnormal expression of non-coding RNAs, such as lncRNA ANRIL, hsa-miR-22-3p, was associated with functional outcome or prognosis in AF patients, and could potentially serve as potential biomarkers for AF-related IS (201, 202). On the basis of current research, it can be expected that new and promising genetics biomarkers for AF-related IS will be further discovered in the near future.

## Summary and Perspective

Biomarkers have become an important integral to the clinical practice of AF. The purpose of this review is to acquaint clinicians and researchers with the progress of biomarkers in AF-related IS. In summary, although a great deal of research has been done on biomarkers for IS prediction in NVAF patients as mentioned above, most potential biomarkers have not yet been translated into clinical use. Nevertheless, these biomarkers can help us to better understand the etiology and pathophysiology of AF-related IS.

An ideal biomarker should be simple, practical, inexpensive, and with high sensitivity. Based on current evidence, we acknowledge that non-paroxysmal AF type, carotid plaque, cardiac troponin, NT-proBNP, and D-dimer are promising biomarkers for IS in NVAF patients since these biomarkers strike a balance between practicality and simplicity. They are easily acquired in clinical practice. Meanwhile, these markers are cardiac-specific or reflecting AF features and pathophysiological processes of stroke. Moreover, the clinical value of these markers has been confirmed by multiple studies.

It is important to recognize, however, that the existing studies have significant limitations. First, most studies are observational studies with small samples size, which limits the clinical value of the identified markers. At the same time, limited by the study design, most studies investigate the correlation between only one biomarker and IS in AF patients, and a single biomarker might be disturbed by other confounding factors, and only a few studies evaluate the role of multiple biomarkers (203). Second, the majority of the study population was treated patients with anticoagulation therapy, and studies focusing on un-anticoagulated patients and other populations are lacking. Third, the end-points of the studies are not uniform, such as IS, systemic TE, TIA, and the combination of them. Additionally, the inclusion criteria and covariates are inconsistent between the studies. Forth, there may be a time-dependent correlation between some biomarkers and IS outcome, and the assessment of a baseline level of the biomarker may not draw a reliable conclusion. For different research investigating the same biomarker, the cut-off values of the biomarker are often incongruent, which makes it impossible to combine the results of the studies. Fifth, the majority of studies merely find the differentially expressed biomarker in AF patients with stroke compared with those without stroke. Almost all the studies fail to show a valuable improvement in clinical usefulness, although a slightly improved predictive performance for IS compared with the commonly used risk score is shown in some studies (Table 2) (204, 205).

**Table 2:** Major verified biomarkers adding in stroke/TE risk stratification beyond CHA_2_DS_2_-VASc score in AF patients.

**Category**	**Biomarker**	**Supportive findings**	**Study population**	**DOI**
ECG markers	Abnormal P-wave Axis	P_2_-CHA_2_DS_2_-VASc score improved the C-statistic for CHA_2_DS_2_-VASc score. In ARIC study: C-statistic was 0.67 vs. 0.60, NRI = 0.25 (0.13, 0.39); In MESA study: C-statistic was 0.75 vs. 0.68 for CHA_2_DS_2_-VASc, NRI = 0.51 (0.18, 0.86).	AF patients	10.1161/CIRCULATIONAHA.118.035411
Cardiac imaging markers	Parameters of LAA shape	LAA shape parameters + CHA_2_DS_2_-VASc score increased the area under the ROC curve from 0.640 to 0.778 (*P* = 0.003).	AF patients	10.1007/s10554-021-02262-8
	LA strain	LA strain had an incremental value over the CHA_2_DS_2_-VASc score (*P* <0.0001).	AF patients	10.1016/j.echo.2014.03.010
	Video intensity value of LASEC	Video intensity value of LASEC had better performance than CHA_2_DS_2_-VASc (0.844 ± 0.041 vs. 0.720 ± 0.065).	NVAF patients	10.1038/srep27650
	Left ventricular relative wall thickness	CHA_2_DS_2_-VASc + RWT increased the area under the ROC curve from 0.614 (0.5734–0.6562) to 0.624 (0.5823–0.6667), NRI = 0.25 (0.11–0.40).	NVAF patients	10.1093/ehjqcco/qcaa003
Atherosclerotic markers	cIMT, carotid plaque	C-statistics increased from 0.648 (95% CI, 0.538–0.757) to 0.716 (95% CI, 0.628–0.804) in the CHA_2_DS_2_-VASc score model after the addition of cIMT and carotid plaque as a vascular component (*P* = 0.013).	AF patients	10.3904/kjim.2019.099
	cIMT, carotid plaque	The addition of cIMT+plaque to the CHA_2_DS_2_-VASc score marginally increased the C-statistic from 0.685 (0.623–0.747) to 0.698 (0.638–0.759).	AF patients	10.1161/STROKEAHA.116.013133
Cardiac biomarkers	NT-proBNP	The addition of NT-proBNP to the CHA_2_DS_2_-VASc score increased the C-statistic from 0.62 (0.59–0.65) to 0.68 (0.56–0.71), NRI = 0.174 (*P* = 0.047).	AF patients	10.1161/STROKEAHA.113.003338
	NT-proBNP, cTnI	CHA_2_DS_2_-VASc + cTnI + NT-proBNP increased the C-statistic from 0.68 to 0.72 (*P* <0.0001).	AF patients	10.1161/CIRCULATIONAHA.111.038729
	NT-proBNP	Adding NT-proBNP levels to the CHA_2_DS_2_-VASc score improved C-statistics from 0.62 to 0.65 (*P* = 0.0009)	AF patients	10.1016/j.jacc.2012.11.082
	BNP	Adding BNP to the CHA_2_DS_2_-VASc score improved C-statistics from 0.65 (0.56–0.75) to 0.75 (0.67–0.83), NRI = 0.76.	NVAF patients	10.1253/circj.CJ-17-1085
	Troponin, BNP, D-dimer	Combination of biomarkers had better AUROC for the prediction of stroke than CHA_2_DS_2_-VASc (0.378 ± 0.028 vs. 0.410 ± 0.028).	NVAF patients	Int J Health Sci (Qassim), 2019, 13(6): 3-12
	NT-proBNP	Adding NT-proBNP to the CHA_2_DS_2_-VASc score improved C-statistics from 0.624 to 0.666, NRI = 0.180.	AF patients	10.1136/heartjnl-2020-317735
	cTnI, NT-proBNP, D-dimer	Adding biomarkers to the CHA_2_DS_2_-VASc score improved C-statistics from 0.586 (0.565–0.607) to 0.708 (0.688–0.728), NRI = 0.594 (*P* <0.001).	AF patients	10.1001/jamacardio.2016.3311
	cTnT	Adding cTnT to the CHA_2_DS_2_-VASc score improved the C statistic from 0.620 to 0.635 (*P* = 0.0226).	AF patients	10.1016/j.jacc.2013.07.093
Routine blood test markers	NLR	Adding NLR to the CHA_2_DS_2_-VASc score increased the AUC from 0.627 (0.612–0.643) to 0.635 (0.619–0.651).	AF patients	10.1111/jth.13006
	MPV, D-dimer	The addition of MPV and D-dimer to the CHA_2_DS_2_-VASc score increased the C-statistic from 0.761 to 0.816.	NVAF patients	10.1186/s12872-020-01525-x
Lipid markers	LDL-C	AUCs for CHA_2_DS_2_-VASc score and CHA_2_DS_2_-VASc score plus LDL-C were 0.591 and 0.674.	NVAF patients	10.1016/j.amjcard.2016.12.031
	LDL-C/HDL-C ratio	AUC of the CHA_2_DS_2_-VASc score plus LDL-C/HDL-C was higher than that of the CHA_2_DS_2_-VASc score (0.91 vs. 0.89, *Z* = 3.26, *P* <0.01).	NVAF patients	10.1186/s12944-020-01392-7
Genetic markers	Genetic variants	Compared with CHA_2_DS_2_-VASc, the integrated tool improved net reclassification (NRI = 2.3%).	AF patients	10.1161/CIRCGEN.120.003168
Urine markers	Urine albumin	AUC of CHA_2_DS_2_-VASc-UA_2_ score was larger than that of CHA_2_DS_2_-VASc score (0.873 vs. 0.860, *P* <0.01).	NVAF patients	10.1016/j.ijcard.2016.07.145

*TE, thromboembolism; AF, atrial fibrillation; ECG, electrocardiogram; NRI, net reclassification improvement; LAA, left atrial appendage; LA, left atrium; LASEC, left atrial spontaneous echo contrast; NVAF, non-valvular atrial fibrillation; RWT, relative wall thickness; ROC, receiver operating characteristic curve; cIMT, carotid intima-media thickness; NT-proBNP, N-terminal prohormone of brain natriuretic peptide; cTnI, cardiac troponin I; BNP, B-type natriuretic peptide; AUROC, area under the receiver operating characteristic curve; cTnT, cardiac troponin T; NLR, neutrophil-to-lymphocyte ratio; MPV, mean platelet volume*.

Incorporating biomarkers into existing models may allow improved predictive accuracy and guide individualized anticoagulation treatment, but it brings complexity. Given these uncertainties, what should the clinician do? First, we believe it is still necessary to discover novel biomarkers and verify the predictive value of current markers in large-scale prospective cohort studies. Studies in patients with CHA_2_DS_2_-VASc score of 0–1 are encouraged, thereby improving the prognosis of patients who are not provided with a clear indication for oral anticoagulants in current guidelines. In addition, it is important to find biomarkers that could distinguish between ischemic and hemorrhagic stroke, because most existing indicators in CHA_2_DS_2_-VASc score indicate both bleeding and ischemia. Second, in view of the complexity and interdependence of pathophysiological pathways for AF-related IS, multi-omics and high-throughput analysis should be used to find multiple biomarkers to discover new therapeutic targets. Third, comprehensive studies are needed to integrate current biomarkers and clinical score to optimize the prevention of IS in patients with NVAF. At last, the effectiveness of the “biomarker plus CHA_2_DS_2_-VASc” score-guided treatment strategy in NVAF patients should also be evaluated.

## Author Contributions

LXS, LZ, YKG, HXS, XXZ, and YKB searched literatures and prepared the manuscript. XHZ and BPT provided funds support, conceived the idea, reviewed the drafts, and provided important information for the completion of this manuscript. All authors contributed to the writing and final approval of the manuscript.

## Conflict of Interest

The authors declare that the research was conducted in the absence of any commercial or financial relationships that could be construed as a potential conflict of interest.
